# Two Cases of Heavy Chain MGUS

**DOI:** 10.1155/2016/8749153

**Published:** 2016-04-26

**Authors:** Jan Van Keer, Björn Meijers, Michel Delforge, Gregor Verhoef, Koen Poesen

**Affiliations:** ^1^Nephrology, Dialysis and Renal Transplantation, University Hospitals Leuven, 3000 Leuven, Belgium; ^2^Laboratory of Nephrology, Department of Microbiology and Immunology, KU Leuven, 3000 Leuven, Belgium; ^3^Hematology, University Hospitals Leuven, 3000 Leuven, Belgium; ^4^Laboratory for Stem Cell Biology and Embryology, Department of Development and Regeneration, KU Leuven, 3000 Leuven, Belgium; ^5^Laboratory for Experimental Hematology, Department of Oncology, KU Leuven, 3000 Leuven, Belgium; ^6^Laboratory Medicine, University Hospitals Leuven, 3000 Leuven, Belgium; ^7^Laboratory for Molecular Neurobiomarker Research, Department of Neurosciences, KU Leuven, 3000 Leuven, Belgium

## Abstract

Heavy chain diseases are rare variants of B-cell lymphomas that produce one of three classes of immunoglobulin heavy chains, without corresponding light chains. We describe two patients with asymptomatic heavy chain monoclonal gammopathy. The first patient is a 51-year-old woman with alpha paraprotein on serum immunofixation. The second case is a 46-year-old woman with gamma paraprotein on urine immunofixation. Neither patient had corresponding monoclonal light chains. Workup for multiple myeloma and lymphoma was negative in both patients. These two cases illustrate that heavy chain monoclonal gammopathy can exist in the absence of clinically apparent malignancy. Only a few reports of “heavy chain MGUS” have been described before. In the absence of specialized guidelines, we suggest a similar follow-up as for MGUS, while taking into account the higher probability of progression to lymphoma than to myeloma.

## 1. Introduction

Heavy chain diseases are characterized by the presence of monoclonal heavy chains without light chains in serum and/or urine [1]. They are rare variants of B-cell lymphomas that produce one of three classes of immunoglobulin heavy chains: alpha, gamma, or mu [2–4]. The clinical manifestations vary with the heavy chain isotype and range from an asymptomatic presentation to aggressive lymphoma [5]. In this report, we describe two patients with asymptomatic heavy chain monoclonal gammopathy, who meet the criteria of “monoclonal gammopathy of undetermined significance” (MGUS) [6].

## 2. Case 1

A 51-year-old woman with a past medical history of depression and dyslipidemia was referred by her general practitioner to the hematology consultation. During the workup of myalgia after start of rosuvastatin therapy, an abnormal serum protein electrophoresis was discovered. At the time of referral, rosuvastatin was stopped and myalgia had disappeared. The patient was asymptomatic. Her only medication consisted of risperidone 1 mg. She never smoked. Physical findings were normal. Laboratory findings included hemoglobin of 14.0 g/dL, total leukocyte count of 8.4 × 10^9^/L with normal differential count, and platelets of 360 × 10^9^/L. Serum creatinine was 0.62 mg/dL (eGFR > 90 mL/min/1.73 m^2^) and blood urea nitrogen 10 mg/dL. Calcium level was normal. Liver function, lactate dehydrogenase, creatine kinase, and coagulation tests were within normal limits. C-reactive protein was 1.6 mg/L (reference value < 5 mg/L).

**Figure 1:**
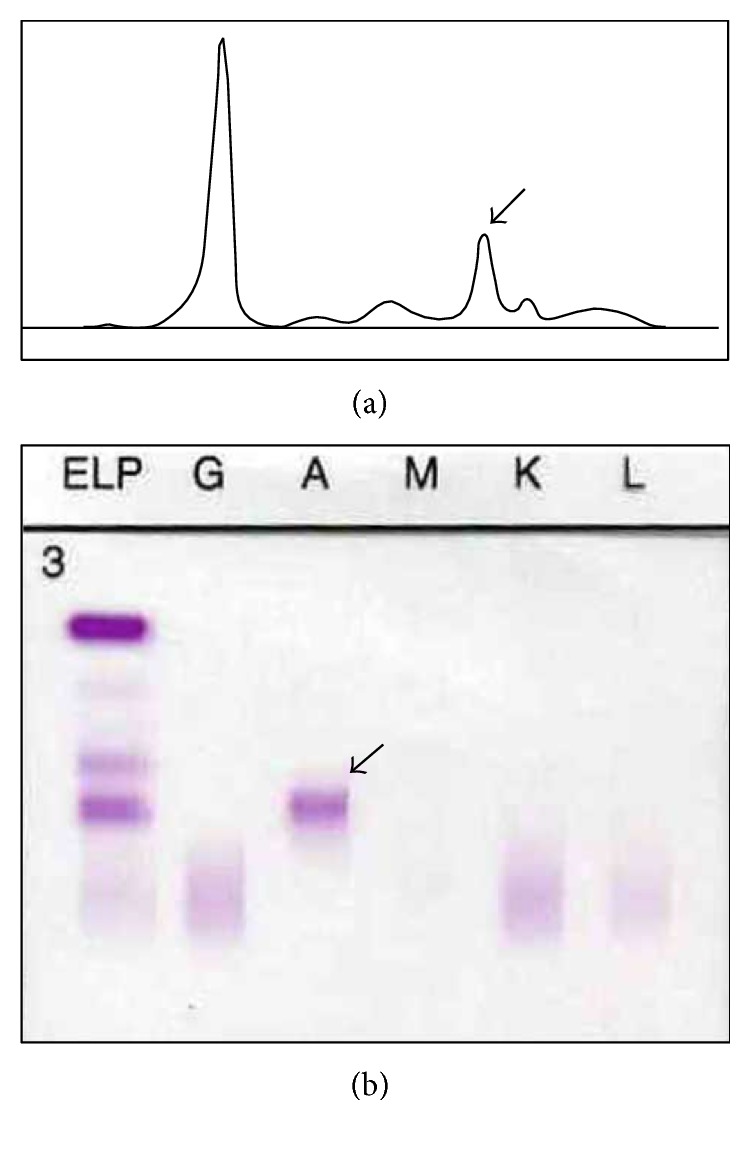
(a) Serum protein electrophoresis showing monoclonal spike in the beta-fraction (arrow). (b) Serum immunofixation showing presence of alpha paraprotein (arrow), without corresponding kappa or lambda light chains.

Serum protein electrophoresis showed a monoclonal spike of 8.9 g/L in the beta-fraction (see Figure 1). Immunofixation revealed an alpha paraprotein, without corresponding monoclonal kappa or lambda chains. 24-hour urine collection contained 0.11 g of protein, without monoclonal spike. Serum total IgA level was 7.20 g/L.

Bone marrow was normocellular with trilineage hematopoiesis and normal percentage of plasma and lymphoid cells. X-ray metastatic bone survey was negative. Chest X-ray and abdominal ultrasound showed no lymphadenopathy or organomegaly. Upper gastrointestinal endoscopy excluded MALT lymphoma. During 8 years of follow-up, the patient developed hypertension and type 2 diabetes; otherwise she remained asymptomatic. Kidney function remained stable. Serum monoclonal spike rose from 8.9 to 10.9 g/L and total IgA from 7.20 to 9.53 g/L (see Table 1). Urine electrophoresis stayed negative.

In conclusion, we describe an asymptomatic patient with an incidental finding of monoclonal gammopathy, corresponding to alpha heavy chains without monoclonal light chains. As workup for lymphoma, myeloma, and monoclonal gammopathy related end organ damage was negative, we diagnosed a heavy chain monoclonal gammopathy of undetermined significance (MGUS). During 8 years of follow-up, serum monoclonal spike was relatively stable and urine electrophoresis remained negative.

## 3. Case 2

A 46-year-old woman was referred to the nephrology consultation for the investigation of chronic kidney disease, with serum creatinine of 1.72 mg/dL (eGFR: 35 mL/min/1.73 m^2^) and blood urea nitrogen of 66 mg/dL. The patient's medical history included squamous cell carcinoma of the right ovary, for which she had undergone extensive surgery (total pelvic exenteration with colostomy and Bricker diversion, sigmoid resection, and partial enterectomy), chemotherapy (cisplatinum, 5-fluorouracil, and mitomycin C, followed by carboplatin, etoposide, ifosfamide, and paclitaxel), and pelvic radiotherapy. The remainder of her past medical history was remarkable for hypertension, anemia of chronic kidney disease, and short bowel syndrome after the aforementioned surgery, for which she was treated with total parenteral nutrition. Her medication included darbepoetin alfa 60 *µ*g per 2 weeks, ramipril 5 mg, barnidipine 10 mg, sodium bicarbonate 4 g daily, and an estradiol patch. She never smoked. Blood pressure was well controlled.

**Figure 2:**
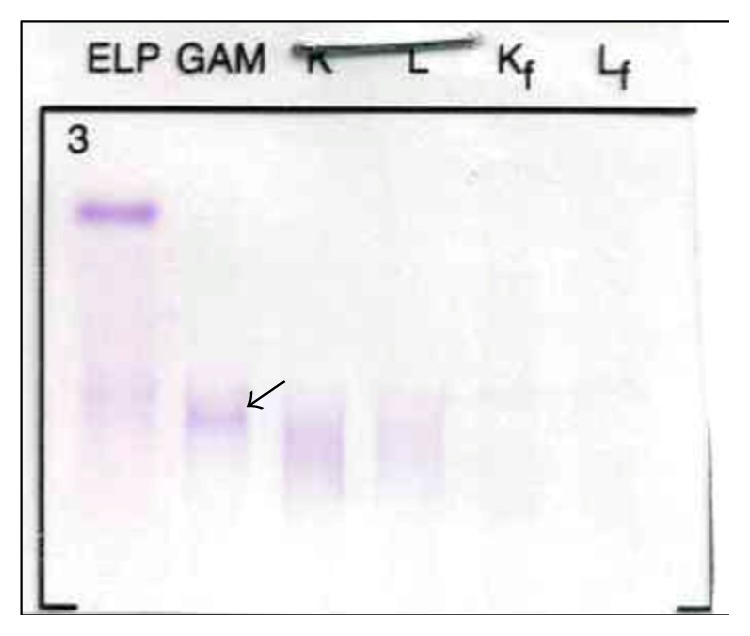
Urinary immunofixation shows a gamma paraprotein (arrow), without corresponding monoclonal light chains.

Serum and urine protein electrophoresis and immunofixation were ordered in the workup of chronic kidney disease. Immunofixation of the 25-fold concentrated 24-hour urine collection showed the presence of a weak monoclonal band typed as gamma heavy chain, without monoclonal kappa or lambda immunoglobulin light chains (see Figure 2). Serum electrophoresis and immunofixation were normal. Total IgG, IgA, and IgM values were 14.40 g/L, 2.73 g/L, and 0.78 g/L, respectively. Serum free light chains were elevated in accordance with the degree of renal insufficiency: 35.00 mg/L for kappa and 35.80 mg/L for lambda light chains, with a normal kappa/lambda ratio (0.98).

Hemogram showed a hemoglobin of 11.2 g/dL, total leukocyte count of 5.14 × 10^9^/L with a normal differential count, and platelets of 125 × 10^9^/L. Peripheral blood smear showed no abnormal lymphocyte population. Liver function, coagulation tests, lipid profile, and fasting and postprandial blood sugars were within normal limits. C-reactive protein was 1.9 mg/L (reference value, < 5 mg/L). Urine sediment showed sterile pyuria and microscopic hematuria (48/*µ*L), a normal finding given the Bricker urinary diversion. Proteinuria on a 24-hour urine collection was 0.23 g.

Bone marrow biopsy showed normal presence of all cell lines with a mild elevation of plasma cells (3.7%), without an atypical morphology. Whole body computed tomography (CT) with 18-fluorodeoxyglucose (FDG) positron emission tomography (PET) showed no evidence of lymphoma. Ultrasound examination revealed relatively small kidneys (left: 99 mm, right: 94 mm) with bilateral significant dilatation of the pyelocaliceal system. Kidney biopsy showed extensive chronic destruction of renal parenchyma in a zonal pattern. All these findings are suggestive of reflux nephropathy. There was no evidence of cast nephropathy, amyloidosis, or heavy or light chain deposition disease on renal biopsy.

In conclusion, we describe a patient with chronic kidney disease due to reflux nephropathy after Bricker urinary diversion, whose urine immunofixation showed presence of monoclonal gamma heavy chains. Workup for lymphoma and myeloma was negative; kidney biopsy showed no monoclonal gammopathy related pathology. Since only one year of follow-up is available, early stage Franklin's disease cannot be entirely excluded. No specific treatment was started. During one year of follow-up the clinical situation remained unchanged.

## 4. Discussion

Heavy chain diseases are B-cell lymphoproliferative disorders that are characterized by the production of monoclonal immunoglobulins consisting of heavy chains without associated light chains [1]. The diagnosis is established by demonstration of alpha, gamma, or mu paraprotein on serum and/or urine protein electrophoresis and immunofixation, without corresponding monoclonal kappa or lambda chains. One should be aware that differing sensitivity of heavy and light chain reagents could potentially cause false negative results of light chain immunofixation. This would incorrectly suggest the diagnosis of heavy chain disease in a case of a regular, intact monoclonal gammopathy.

Alpha, gamma, and mu heavy chain diseases have been described [2–4]. Of these, alpha heavy chain disease (Seligmann's disease) is the most common. It was first described in 1968 by Seligmann et al. [7], hence its eponym. Approximately 175 cases have been described. The patients typically present with chronic diarrhea, malabsorption, and weight loss. Some patients develop extensive mesenteric adenopathy [2]. Alpha heavy chain disease is also known as immunoproliferative small intestinal disease. It is a form of MALT lymphoma and is associated with poor sanitation and* Campylobacter jejuni* infection [8]. Some cases may respond to treatment with antibiotics. Therefore an endoscopic and microbiologic study of the digestive tract is indicated in the workup of alpha heavy chain disease.

Gamma heavy chain disease is intermediate in frequency. It is also called Franklin's disease [9], after the author of the first report in 1964. Since then, approximately 130 cases have been described in the literature. The classical presentation is one with generalized lymphadenopathy, splenomegaly, and anemia [10]. The most distinctive symptom is palatal edema resulting from enlargement of nodes in Waldeyer's ring, sometimes leading to respiratory compromise [2]. Gamma heavy chain disease is associated with autoimmune disease in almost one-third of cases: rheumatoid arthritis is most common; other associated diseases include autoimmune hemolytic anemia and idiopathic thrombocytopenic purpura [1].

Mu heavy chain disease is the least common of the heavy chain diseases. Less than 30 cases have been reported. Its clinical features resemble chronic lymphocytic leukemia. Vacuolated lymphocytes in the bone marrow are characteristic [11]. Unlike alpha and gamma heavy chain diseases, mu heavy chain disease can be associated with increased free light chain excretion as some tumor cells seem to have a defect in the assembly of both light and heavy chains.

We described two cases (one alpha and one gamma) of heavy chain monoclonal gammopathy who were asymptomatic. Based the low value of the monoclonal spike (<30 g/L) and bone marrow monoclonal plasma cells (<10%), absence of end organ damage, and clinical or radiographic evidence of lymphoma, these cases met the criteria for monoclonal gammopathy of undetermined significance (MGUS) [6].

Only a few reports of “heavy chain MGUS” have been described previously [1, 11–15]. There are no established guidelines for follow-up. We propose the same follow-up as for normal MGUS. However, unlike normal MGUS, there are no validated risk factors for progression that could guide intensity of follow-up. Note that most heavy chain diseases are lymphoproliferative disorders. Therefore, heavy chain MGUS is more likely to progress to lymphoma than to myeloma. Follow-up should focus on assessment of lymphadenopathy and organomegaly. This can be done by regular physical examination in most cases and imaging on indication. Follow-up should include measurement of serum and urinary M-protein, complete blood count, creatinine, and electrolytes. M-protein level can be determined semiquantitatively by protein electrophoresis and immunofixation. At this moment, there are no commercially available quantitative techniques to measure monoclonal free heavy chains.

## 5. Conclusion

Heavy chain diseases are rare variants of B-cell lymphomas characterized by the presence of monoclonal heavy chains, alpha, gamma, or mu, without corresponding light chains. Clinical presentation is diverse. We describe two asymptomatic cases that meet the criteria of MGUS. This report demonstrates that heavy chain monoclonal gammopathy can exist in the absence of clinically apparent malignancy, similar to other forms of MGUS. In the absence of specialized guidelines, we suggest a similar follow-up as for MGUS, while taking into account the higher probability of progression to lymphoma than to myeloma.

## Figures and Tables

**Table 1:** Evolution of total IgA and estimated monoclonal spike during 8 years of follow-up.

Time (y)	IgA (g/L)	Estimated monoclonal spike (g/L)
0	7.20	8.9
1	6.72	NA
2	7.08	NA
3	8.18	9.1
4	8.63	NA
5	7.72	NA
6	NA	NA
7	NA	NA
8	9.53	10.9
